# Detecting pathological features and predicting fracture risk from dual-energy X-ray absorptiometry images using deep learning

**DOI:** 10.1016/j.bonr.2021.101070

**Published:** 2021-04-24

**Authors:** Tomi Nissinen, Sanna Suoranta, Taavi Saavalainen, Reijo Sund, Ossi Hurskainen, Toni Rikkonen, Heikki Kröger, Timo Lähivaara, Sami P. Väänänen

**Affiliations:** a Department of Applied Physics, University of Eastern Finland, POB1627, 70211 Kuopio, Finland; b Department of Clinical Radiology, Kuopio University Hospital, POB1777, 70211 Kuopio, Finland; c Institute of Clinical Medicine, University of Eastern Finland, POB1627, 70211 Kuopio, Finland; d Department of Orthopaedics, Traumatology and Hand Surgery, Kuopio University Hospital, POB1777, 70211 Kuopio, Finland

**Keywords:** Deep learning, Dual-energy X-ray absorptiometry, Degeneration, Fracture risk, Lumbar spine, Scoliosis, Trabecular bone score

## Abstract

Dual-energy X-ray absorptiometry (DXA) is the gold standard imaging method for diagnosing osteoporosis in clinical practice. The DXA images are commonly used to estimate a numerical value for bone mineral density (BMD), which decreases in osteoporosis. Low BMD is a known risk factor for osteoporotic fractures. In this study, we used deep learning to identify lumbar scoliosis and structural abnormalities that potentially affect BMD but are often neglected in lumbar spine DXA analysis. In addition, we tested the approach's ability to predict fractures using only DXA images. A dataset of 2949 images gathered by Kuopio Osteoporosis Risk Factor and Prevention Study was used to train a convolutional neural network (CNN) for classification. The model was able to classify scoliosis with an AUC of 0.96 and structural abnormalities causing unreliable BMD measurement with an AUC of 0.91. It predicted fractures occurring within 5 years from the lumbar spine DXA scan with an AUC of 0.63, meeting the predictive performance of combined BMD measurements from the lumbar spine and hip. In an independent test set of 574 clinical patients, AUC for lumbar scoliosis was 0.93 and AUC for unreliable BMD measurements was 0.94. In each classification task, neural network visualizations indicated the model's predictive strategy. We conclude that deep learning could complement the well established DXA method for osteoporosis diagnostics by analyzing incidental findings and image reliability, and improve its predictive ability in the future.

## 1 Introduction

**Fig. 1:**
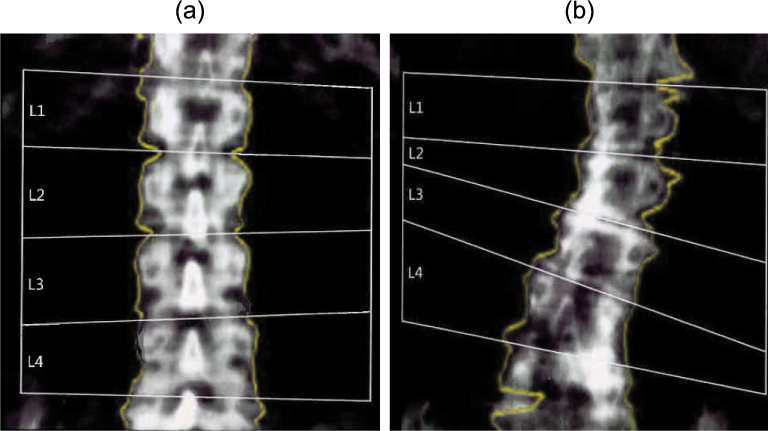
Dual-energy X-ray absorptiometry images of the spine. In DXA measurement, the bone mineral density is calculated for each semi-automatically segmented vertebra (L1-L4). (a) The subject on the left demonstrates a normal lumbar spine. (b) The subject on the right shows scoliosis and severe degenerative changes.

Osteoporosis is a disease of decreased bone mineral density which increases the risk for low energy fractures, particularly in the elderly. Low energy fractures are associated with high morbidity and high mortality. Osteoporosis has been estimated to affect more than 200 million people worldwide (Cooper, 1999). Improving diagnostics may enable early intervention which can affect the patient's quality of life and save costs to society (World Health Organization, 2003). Currently, the diagnosis of osteoporosis is based on measuring bone mineral density (BMD) using Dual-energy X-ray absorptiometry (DXA). DXA scan is performed while the patient lies supine. It uses low-intensity X-ray beams reducing the radiation dose to about one-twelfth compared to conventional lumbar radiography (Messina et al., 2016). This results in lower image quality but sufficient to approximate bone mineral density for the areas of interest (Fig. 1). However, the morbidity of osteoporosis arises not from the loss of bone itself but the associated fractures.

Osteoporotic fractures are fragility fractures, typically caused by a fall from no greater than standing height. These fractures usually occur in the hip, spine, forearm, and humerus (Seeley et al., 1991). Bone mineral density alone is only a moderate predictor of fractures. In fact, most of the patients who sustain fragility fractures do not fall below the osteoporosis BMD threshold defined by the World Health Organization (WHO) (World Health Organization, 2003; Unnanuntana et al., 2010). This has created a demand for more advanced prediction methods that look beyond the bone density.

Several clinical risk factors, independent of BMD, have been identified for fragility fractures. Statistical methods like The Fracture Risk Assessment Tool (FRAX) (Kanis et al., 2008) can be used to predict fracture probability based on variables such as age, fracture history, demographic and physical characteristics, medication, and many lifestyle factors (Unnanuntana et al., 2010). It has been shown that clinical risk factors and BMD together provide better fracture predictions than either alone (Kanis et al., 2007). The prediction models could potentially be improved even further by extracting more fracture risk-related information from the images.

In addition to BMD, DXA images also hold anatomical and pathological information. Scoliosis and degenerative changes are common findings in lumbar spine imaging. Scoliosis is defined as a spinal deformity consisting of lateral curvature and rotation of the vertebrae (Janicki and Alman, 2007). Degenerative changes in the spine are responsible for the loss of normal structure and function. Radiographic features of degeneration include loss of intervertebral disc space, endplate sclerosis, osteophytes, and facet joint arthrosis (Bogduk, 2012). Although DXA imaging is not used for diagnosing scoliosis or degeneration in clinical practice, these conditions should be considered when evaluating the scans (American College of Radiology (ACR), 2018). Degenerative changes have been shown to elevate BMD values measured from the lumbar spine (Salo et al., 2014). Scoliosis, in turn, can complicate segmentation and setting of the vertebra levels, which may affect the BMD values (Watts, 2004). These confounders with other abnormalities such as fractures, previous surgery, or artefacts can hamper the analysis and cause discrepancy in DXA interpretation among observers (Hansen et al., 2005). A method taking them into account could support the diagnostics.

To assess the quality of bone, methods extracting additional information from DXA images have been proposed. Hip Structural Analysis (HSA) (Beck et al., 1990) measures geometric parameters associated with bone strength whereas Trabecular Bone Score (TBS) (Pothuaud et al., 2008) analyzes grey-level variations in bone texture claimed to reflect bone microarchitecture. These methods have been shown to serve as independent predictors of fracture (Maquer et al., 2016; LaCroix et al., 2010; Pulkkinen et al., 2004). However, their clinical relevance and ability to improve fracture prediction are not completely clear (Maquer et al., 2016; Kaptoge et al., 2008). A possible limitation is that they focus on very specific features instead of analyzing the bone as a whole. In addition to the known factors, the images may contain visual evidence of fracture risk that is not yet known, or even too difficult for the human eye to detect.

Deep learning is a set of machine learning techniques that can automatically discover the features needed to predict certain outcomes from data. It uses deep artificial neural networks that have multiple layers between the input and output (Bengio, 2009). The last layers learn complicated concepts by building them out of simpler ones identified in the first layers. The key aspect of deep learning is that these layers are not designed by human engineers. Instead, they are learned from data using a learning procedure such as supervised learning. In supervised learning, a high number of input samples with known outcomes are fed into the network. The network learns the relationship between input and output by iteratively predicting and adjusting its connective weights according to prediction error. Once trained, the network produces deterministic predictions from new input data in real-time (Goodfellow et al., 2016).

A commonly recognized challenge in neural networks has been their poor interpretability. The model's predictive strategy is often unclear failing to provide explanations on the predictions. This has led to the emerge of neural network visualization as its own branch in machine learning research (Simonyan et al., 2014; Erhan et al., 2009). Visualization methods can reveal the shape and size of the extracted features, as well as their relative importance to the prediction. Improving the transparency of prediction models can help build trust in machine learning based diagnostics.

Deep learning has produced promising results in medical image analysis including detection of scoliosis (Horng et al., 2019; Yang et al., 2019) and degenerative changes in different bone sites (von Schacky et al., 2020; Tiulpin et al., 2018) from other image modalities than DXA. Many studies have exploited conventional radiography, magnetic resonance imaging, and computed tomography (CT) (Cabitza et al., 2018; Burns et al., 2020). Automated fracture risk assessment based on CT of the chest has been proposed as an alternative to FRAX analysis (Dagan et al., 2020). Full body DXA images have been used to identify scoliosis in children (Jamaludin et al., 2020) whereas lateral DXA images of the lumbar spine have been used to identify existing vertebral fractures (Derkatch et al., 2019).

We investigate the use of deep learning to identify scoliosis, detect BMD measurement unreliability caused by structural abnormalities, and predict fractures from DXA images of the lumbar spine. Furthermore, we aim to explain the models' predictive strategy in each classification task through visualizations.

## 2 Material and methods

### 2.1 Data

The primary research material was collected by Kuopio Osteoporosis Risk Factor and Prevention Study (OSTPRE) (Rikkonen et al., 2010) and its side study OSTPRE Fracture Prevention Study (OSTPRE-FPS) (Salovaara et al., 2010). OSTPRE is a population-based prospective cohort study aimed to investigate factors associated with bone mineral density, bone loss, falls, and fractures in peri- and postmenopausal women. It originally included 14,220 women aged 47 to 56 years who lived in Kuopio region in February 1989. Every five years, a subset of the patients have also undergone DXA scans.

**Fig. 2:**
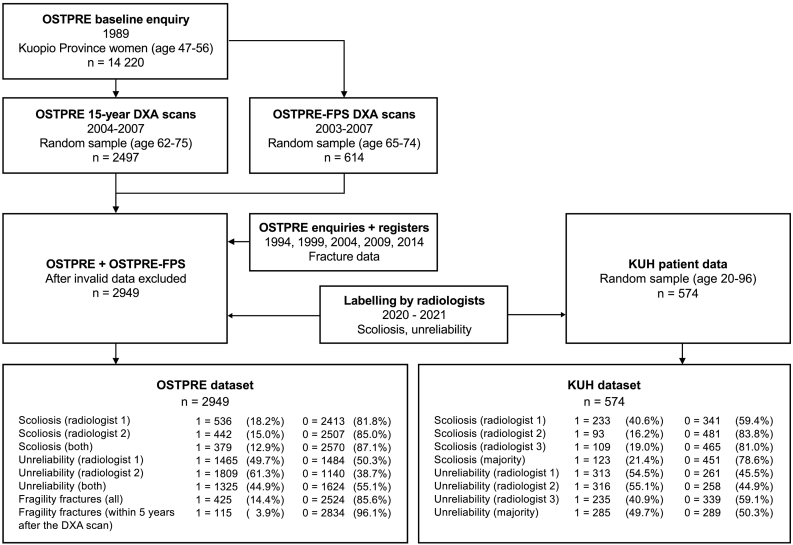
Data selection flowchart. The OSTPRE study originally involved 14,220 elderly females from Kuopio region. 2497 participants from the OSTPRE study and 614 non-overlapping participants from a side-study OSTPRE-FPS had DXA scans taken between 2003 and 2007. These subsets were combined and linked with the fracture data from postal enquiries in the years 1994, 1999, 2004, 2009, and 2014. The fractures reported by the participants were verified from the national patient registers. Some participants were excluded due to lost or corrupted measurement data. The DXA images of the final dataset were labelled for scoliosis and unreliability by two radiologists. This resulted in 2949 labelled DXA-images in the OSTPRE dataset. An external non-overlapping dataset of 574 random patients was gathered from Kuopio University Hospital (KUH) and labelled for scoliosis and unreliability by three radiologists. The number and proportion of positive (1) and negative (0) samples in each case are shown in the final dataset boxes.

We used the DXA images from OSTPRE 15-year follow-up measurements from 2004 to 2007 and OSTPRE-FPS measurements from 2003 to 2007 (Fig. 2). They were produced with a Lunar Prodigy DXA scanner (GE Healthcare, Madison, WI) using a pixel size of 1.05 × 0.60 mm (height x width) and an effective radiation dose of 4 μSv. Measured X-ray attenuation was calibrated daily using a BMD phantom to match g/cm^2^ values of hydroxyapatite. The BMD values were measured for the lumbar spine and hip according to standard procedures of the device. The BMD T-scores, meaning the number of standard deviations (SD) from the reference mean, were calculated according to Finnish female population reference values (hip mean: 0.98 g/cm^2^, hip SD: 0.12 g/cm^2^, lumbar spine mean: 1.20 g/cm^2^, lumbar spine SD: 0.12 g/cm^2^) (Kröger et al., 1992). In clinical use, the DXA device produces a report including the segmented images and measured BMD values. With the help of the device provider GE Healthcare, we exported the pixel-wise DXA-images from the scanner in MATLAB binary format.

The OSTPRE dataset consisted of 2949 female patient cases. At the time of imaging, the average age of the patients was 68 years varying from 62 to 75 years with a standard deviation (SD) of 2 years. The fracture data were collected in the follow-up questionnaires in 1994, 1999, 2004, 2009, and 2014. The fragility fractures were identified based on the site of the fracture (lumbal spine, thoracal spine, proximal humerus, distal forearm, and proximal femur). Clear cases of high energy fractures, like the ones caused by traffic accidents, were excluded. All the fractures reported by the participants were verified from the patient records. Also, a complete history for hip fractures was checked from the national registers, but other types of fracture depended on the patient's own reporting (Honkanen et al., 1999). To make the classification task binary, the count of fractures was discarded, and the output variable was defined merely as occurrence. We defined two different fracture labels. One stating whether the patient had suffered a fragility fracture at some point during the 25-year follow-up period from the start of the OSTPRE study (14.4% positives). This label, indicating a realized risk of fracture, was used in training the network. For validating the network's ability to predict future fractures, we defined another label stating whether the patient had suffered a fracture within 5 years after the scan (3.9% positives). This approach of two labels enabled a higher positive rate to boost the training while still being validated in the prediction of future fractures.

All the DXA images were visually evaluated by two radiologists. They agreed on the medical standards on which the labels were based, but performed the actual work independently. Scoliosis was labelled by the visual estimation of lateral curvature as positive (Cobb angle more than 10°) or negative (no scoliosis or Cobb angle less than 10°). The inter-rater agreement in scoliosis labelling was 92.5% (Cohen's kappa *k* = 0.73). The label for the unreliability of BMD measurement was based on the level of structural changes that might compromise the BMD analysis. According to the American College of Radiology (ACR) (American College of Radiology (ACR), 2018) and the International Society for Clinical Densitometry (ISCD) (The International Society for Clinical Densitometry (ISCD), 2019) recommendations, DXA interpretation should be based on a minimum of two (regular) vertebrae. Therefore, a sample was classified as positive if it had 3–4 vertebra between L1-L4 affected by degeneration or other focal structural abnormalities such as fractures, previous surgery, or artefacts. Any degenerative changes that visibly affected X-ray attenuation, vertebral boundaries, or disk space were noted. In unreliability labelling, the radiologists reported a significant number of borderline cases, which was reflected in the moderate inter-rater agreement of 78.8% (*k* = 0.58). For training and evaluation of the deep learning models, we combined the annotations of the two radiologists. Only the cases with agreed positive labels between both radiologists were considered positive and all other negative.

For the assessment of generalizability, we gathered an external dataset of 574 random patients from Kuopio University Hospital (KUH). Ethical permission was granted by Ethics Committee of Hospital District of Northern Savo (1298/13.02.00/2019). These images had been produced with a different device of the same type operated by different staff compared to the OSTPRE dataset. It had no overlapping patients and no preselected age group or gender. 459 (80%) of the patients were female and 115 (20%) were male. The average age was 64 years ranging from 20 to 96 years with a standard deviation of 10 years. Fracture information was not available for this dataset, but three radiologists labelled it for scoliosis and unreliability by the same labelling principles. Two of these radiologists were the same as in OSTPRE dataset labelling. In this external dataset, the combined labels were based on the majority opinion among the three experts. The inter-rater agreements in scoliosis labelling among the pairs were 75.6% (*k* = 0.44) for radiologist 1 and radiologist 2, 92.3% (*k* = 0.74) for radiologists 2 and 3, and 78.4% (*k* = 0.51) for radiologists 1 and 3. In unreliability, the respective inter-rater agreements were 80.7% (*k* = 0.61), 79.3% (*k* = 0.59), and 76.3% (*k* = 0.53). To estimate the intra-rater reliability, the radiologists labelled the same set of images again after some days. In scoliosis, the intra-rater reliabilities were 92.9% (*k* = 0.85) for radiologist 1, 94.1% (*k* = 0.79) for radiologist 2, and 94.6% (*k* = 0.82) for radiologist 3. In unreliability, they were 89.2% (*k* = 0.78), 85.9% (*k* = 0.71), and 86.6% (*k* = 0.71), respectively.

### 2.2 Deep learning approach

Convolutional neural network (CNN) was chosen as the deep learning model. It has been used for a wide variety of image analysis applications including face recognition (Taigman et al., 2014) systems, self-driving cars (Hadsell et al., 2009), and segmentation of microscopic images (Ning et al., 2005). CNN is based on an artificial neural network consisting of multiple layers of neurons that have learnable weights. In addition, it introduces three architectural ideas: local receptive fields, shared weights, and spatial downsampling (Lecun and Bengio, 1995). With local receptive fields, neurons can extract basic visual features such as edges, endpoints, and corners. These features are then combined in the following layers. To simplify the model, similar feature detectors with shared weights are used across the entire image. This limits the capacity of the network, but more importantly, improves its generalization ability. The set of weight sharing units is called a convolutional kernel. Each kernel produces a feature map as an output. Pooling layers are used to downsample these feature maps by summarizing the presence of features in certain areas. This makes the model more robust to changes in the exact position of the features in the image (Lecun et al., 2015).

Generating a neural network model that works for certain data is highly experimental. The best performing architecture is case dependent and finding the parameters is often by trial and error (Curry and Morgan, 2006; Bergstra and Bengio, 2012). Automated hyperparameter tuning is the set of processes to find the best performing parameters of a model automatically. We utilized two of such processes: Random search (Bergstra and Bengio, 2012) and Hyperband (Li et al., 2018). Random search repeatedly picks the parameter values randomly from predefined ranges, trains the model, and evaluates its performance. Hyperband is a variation of random search that introduces a form of early stopping for bad runs. This enables allocating computational resources for more promising parameter combinations. In this study, the initial search for sufficient depth (number of layers) and width (number of kernels) of the network architecture was performed by automated tuning. Further adjustments were made based on manual experiments, which are described in more detail in the supplementary material. Experimentation on different architectures for the classification tasks resulted in a conclusion that the model is determined primarily by the input data rather than the output label. To demonstrate that CNN can extract diverse features without task-specific modification we used the same architecture and hyperparameters for all three classification tasks.

**Fig. 3:**
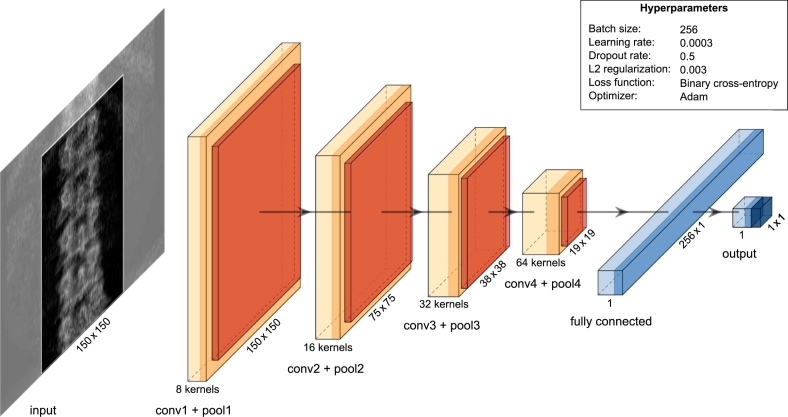
Deep learning architecture overview. The grayscale DXA images are preprocessed and automatically cropped to 150 × 150 pixels before feeding as input for the convolutional neural network. Convolutional layers (conv1-conv4) with pooling layers (pool1-pool4) extract features of different abstraction levels gradually reducing the size of the feature maps. The extracted features are passed on to classification by a fully connected layer. The output layer of the network is a single neuron producing the classification output value between 0 and 1. The Number of kernels is shown below each layer and the size of the resulting feature map next to each layer.

The final model consisted of 4 convolutional layers, 4 pooling layers, and 1 fully connected layer (Fig. 3). The raw images of 300 pixels in width and 150–178 pixels in height were automatically cropped to an input size of 150 × 150 pixels. This was done by searching the centre line of the spine and including 75 pixels on both sides to the final input image (see supplementary material for details). The training data was augmented by generating horizontally flipped versions of the original images. All the convolutional layers used a kernel size of 5 × 5 pixels, ‘same’ padding, and rectified linear unit (ReLU) (Nair and Hinton, 2010) activation function. Downsampling was performed by a max-pooling layer, with a pooling size of 2 × 2 pixels, after each convolutional layer. The output from the last pooling layer was flattened and fed to a fully connected layer for classification. L2 regularization (Tikhonov and Glasko, 1965) was applied on all the convolutional layers and Dropout regularization (Srivastava et al., 2014) on the fully connected layer. The output layer contained a single neuron with a sigmoid activation function to produce the prediction output in the range of 0 to 1.

To address the challenge of transparency, we utilized several visualization techniques suited for convolutional neural networks. They enabled us to qualitatively assess the performance of our models. Heatmaps highlighting the important regions of the image for prediction were generated by Gradient-weighted Class Activation Mapping (Grad-CAM) (Selvaraju et al., 2017). The method uses the gradients of the target class flowing into the last convolutional layer to produce a coarse localization heatmap. Vanilla gradients method (Simonyan et al., 2014) was employed to indicate the shapes and textures the network extracts. It uses backpropagation going all the way to the input layer to track which pixels need to be changed the least to affect the output value the most. Such pixels can be expected to correspond to the objects recognized by the prediction model.

The previously mentioned methods depict the prediction process of the whole network. To visualize the function of specific layers and kernels we used two different approaches. One is to render the feature maps for a single input sample (Yosinski et al., 2015). This gives a rough view on how the neural network sees the image in different phases of feature extraction. The method is very simple but informative since the output of a kernel is rendered as it is. Another approach is to generate an artificial input sample by using the activation maximization method (Erhan et al., 2009). An input sample is initialized with random noise and fed to the trained network with the target class label. As the sample most likely produces a prediction error, the gradients for the kernels are calculated like in the training phase. But, instead of changing the weights of the kernel, the input image is transformed towards a better prediction result. As we repeat these steps, the image begins to take the form that maximizes the activation of the convolutional kernel. This visualization can indicate the purpose and variability of the learned kernels, irrespective of the input image. The kernels in the last convolutional layers usually extract human-recognizable features and are therefore the most interesting to visualize (Erhan et al., 2009; Lecun et al., 2015).

The deep learning models were built using Python 3.6 and the GPU supported version of TensorFlow 2.1 (Abadi et al., 2015) machine learning framework. Data selection and preprocessing, statistical analysis, and graphs were implemented using scikit-learn 0.19.1 (Pedregosa et al., 2011), Statsmodels 0.11.1 (Seabold and Perktold, 2010), and Matplotlib 3.1.2 (Hunter, 2007). Keras-Tuner 1.2 (Keras Team, 2019) was used for automated hyperparameter tuning. The experiments were executed in a Linux server equipped with Intel Core i5–6600 3.3GHz CPU, 16GB RAM, and Tesla P100 GPU with 16GB of display memory.

### 2.3 Statistical analysis

The deep learning models were evaluated using 10-fold cross-validation. The OSTPRE dataset was split into 10 random subsets using stratified sampling to retain the same class distribution across all subsets. To obtain predictions for the whole dataset, the model was trained 10 times holding out different subset for validation each time. This process was again repeated 100 times using different random seeds. Mean performance measures and confidence intervals were calculated from the resulting 1000 iterations. The method ensures that, during these iterations, the validation patients are never present in the respective training set. Using repeated 10-fold cross-validation aims at a better estimation of the model skill independent of the split to training and validation samples.

The performance results are reported with accuracy (proportion of correct predictions), the receiver operating characteristic curve (ROC) and the area under the ROC curve (AUC) (Fawcett, 2006), confusion matrix, sensitivity (proportion of correctly identified actual positives), and specificity (proportion of correctly identified actual negatives). The 95% confidence intervals were calculated using Student's *t*-distribution. We show the learning curves by plotting model performance (average error) over training epochs (iterations over the training set). Reviewing the plots can be used to identify problems with learning, such as overfitting or underfitting (Goodfellow et al., 2016). The ROC curves and confusion matrices are shown for the complete dataset from the 10-fold cross-validation producing the median AUC value. The learning curves and visualization results were taken from the cross-validation subset that produced the median AUC value.

In scoliosis and unreliability detection, the models' generalizability was tested with the external KUH dataset. The ten models that produced the median AUC value in the repeated 10-fold cross-validation with the OSTPRE dataset were used to predict on the external test set. We report the mean, minimum, and maximum performance results from these tests.

For benchmarking the deep learning approach in fracture prediction, we built logistic regression models based on one or more predictor variables. The selected variables were the lumbar spine BMD T-score (the minimum of vertebrae L1-L4), the hip BMD T-score (the minimum of the femoral neck or femoral total), the minimum BMD T-score (from spine or hip), TBS (the average of vertebrae L1-L4), and the age of the patient. In addition, the fracture prediction outputs from the deep learning model were included as one variable. This enabled us to analyze the deep learning output's significance when used together with the other predictors.

For classification, the output values produced by the deep learning model needed to be converted to binary labels. The classification boundary in scoliosis and degeneration detection was set to 0.5 representing the model's view on the most probable label for each sample. In fracture prediction, the classes were heavily imbalanced making the negative class almost always more likely. Thus, the threshold was lowered to make the model more sensitive to cases with increased risk, and comparable to the current T-score based diagnostics. The threshold was adjusted to 0.15 to approximately match the sensitivity of the minimum BMD T-score fracture predictor. For the BMD T-score predictors, we used the osteoporosis threshold defined by WHO: 2.5 standard deviations below the reference mean value of a healthy young adult (World Health Organization, 2003).

## 3 Results

### 3.1 Scoliosis detection

**Fig. 4:**
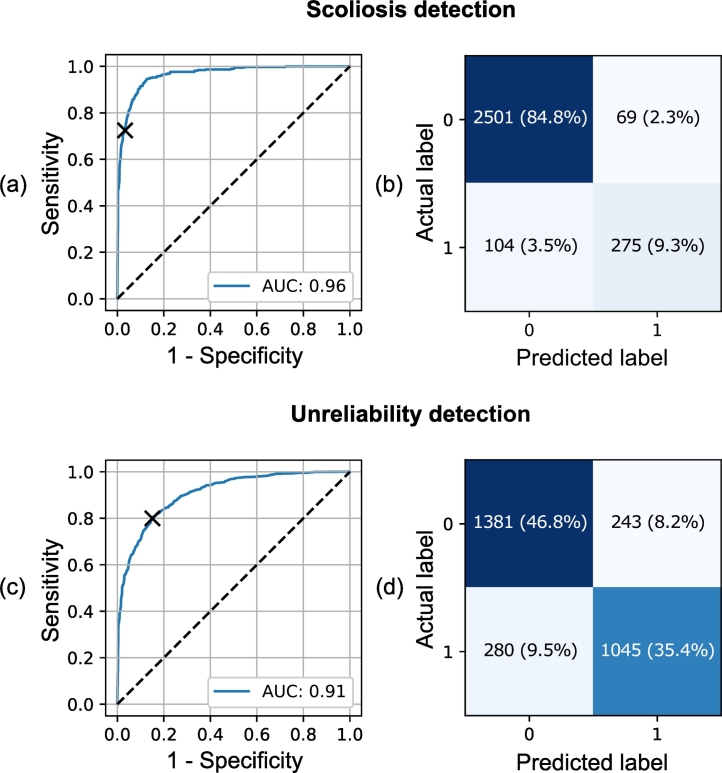
Receiver operating characteristic (ROC) curves with the area under the ROC curve (AUC) values and confusion matrices for (a) and (b) scoliosis detection and (c) and (d) unreliability detection taken from the OSTPRE dataset 10-fold cross-validation with the median AUC performance. The classification boundaries are marked in ROC curves with x-symbols.

In the OSTPRE dataset, the model was able to detect scoliosis with an average AUC of 0.96, accuracy of 94.1%, sensitivity of 70.5%, and specificity of 97.6% (Table 1 and Fig. 4(a) and (b)). The heatmap and gradient visualizations indicate that the model is focusing on the overall curvature of the spine (Fig. 6(a) and supplementary material Fig. 8). This interpretation is supported by the feature maps from the last convolutional layer visualizing vertical contours. Also, the activation maximization method has generated different vertically oriented input patterns. This implies that the kernels have learned to detect bone texture at different angles.

**Table 1:** Repeated 10-fold performance results in scoliosis detection, unreliability detection, and fracture prediction using the OSTPRE dataset. In fracture prediction, also the benchmark results from logistic regression tests are reported. Values shown are averages over 1000 subsets with the 95% confidence intervals in parenthesis.

Experiment	Sensitivity (%)	Specificity (%)	Accuracy (%)	AUC
Scoliosis: Deep learning	70.5 (70.0, 71.0)	97.6 (97.5, 97.6)	94.1 (94.0, 94.2)	0.96 (0.96, 0.97)
Unreliability: Deep learning	78.3 (78.1, 78.6)	85.7 (85.5, 85.8)	82.4 (82.2, 82.5)	0.91 (0.91, 0.91)
Fracture prediction				
Deep learning	60.0 (59.0, 61.0)^a^	58.9 (58.3, 59.4)^a^	58.9 (58.4, 59.4)^a^	0.63 (0.62, 0.65)
Spine T-score (min L1-L4)	58.3 (57.4, 59.1)^b^	59.0 (58.8, 59.1)^b^	58.9 (58.8, 59.1)^b^	0.62 (0.61, 0.64)
Hip T-score (min neck/total)	10.4 (9.9, 11.0)^b^	95.8 (95.8, 95.9)^b^	92.5 (92.4, 92.6)^b^	0.62 (0.60, 0.63)
Min T-score (min spine/hip)	60.0 (59.2, 60.8)^b^	58.2 (58.0, 58.3)^b^	58.2 (58.1, 58.4)^b^	0.63 (0.63, 0.64)
Age	60.0 (59.1, 60.8)^a^	45.5 (45.3, 45.7)^a^	46.0 (45.9, 46.2)^a^	0.55 (0.54, 0.55)
TBS (avg L1-L4)	60.0 (59.2, 60.9)^a^	49.6 (49.4, 49.8)^a^	50.0 (49.9, 50.2)^a^	0.59 (0.58, 0.59)
Min T-score⁎+TBS + Age	60.0 (59.3, 61.0)^a^	61.7 (61.5, 61.9)^a^	61.6 (61.5, 61.8)^a^	0.63 (0.63, 0.64)
Spine T-score⁎+Deep learning⁎	60.0 (59.1, 60.8)^a^	60.9 (60.7, 61.1)^a^	60.9 (60.7, 61.1)^a^	0.64 (0.63, 0.64)
Min T-score⁎+Deep learning	60.0 (59.1, 60.9)^a^	60.4 (60.2, 60.6)^a^	60.4 (60.2, 60.5)^a^	0.64 (0.64, 0.65)
Deep learning⁎+TBS	60.0 (59.1, 61.0)^a^	58.6 (58.4, 58.7)^a^	58.6 (58.4, 58.8)^a^	0.63 (0.62, 0.63)

a Result acquired with the classification boundary set to approximately match the sensitivity of the minimum BMD T-score fracture predictor.

b Result acquired with the classification boundary set to the osteoporosis T-score threshold of −2.5 SD (World Health Organization, 2003; Kröger et al., 1992).

⁎ Predictor variable remained statistically significant (*p* < 0.05) in the logistic regression model.

The learning curves (Fig. 7(a)) show that the model has sufficient capacity to learn from the training data. The training error drops steeply at first and then steadily approaches zero. The validation error follows the training curve before the model starts to slowly overfit after 40 epochs of training.

The models tested with the external KUH dataset produced an average AUC of 0.93 (range 0.92–0.95) (Table 2). Accuracy in the test set was 89.6%, sensitivity 74.5%, and specificity 93.7%.

**Table 2:** External test performance results in scoliosis and unreliability detection using the KUH dataset. Values shown are averages with minimum and maximum value over the 10 models that produced the median AUC value in repeated 10-fold cross-validation using the OSTPRE dataset.

Experiment	Sensitivity (%)	Specificity (%)	Accuracy (%)	AUC
Scoliosis	74.5 (65.0–80.5)	93.7 (90.9–96.9)	89.6 (88.3–90.2)	0.93 (0.92–0.95)
Unreliability	92.7 (89.1–97.9)	72.9 (56.7–81.0)	82.8 (77.2–85.0)	0.94 (0.94–0.95)

### 3.2 Unreliability detection

In detecting unreliability of BMD measurement in the OSTPRE dataset, the model produced an average AUC of 0.91, accuracy of 82.4%, sensitivity of 78.3%, and specificity of 85.7% (Table 1 and Fig. 4(c) and (d)). The heatmap visualizations have notable variation between test samples (Fig. 6(b) and supplementary material Fig. 9), but a common factor seems to be the focus on individual vertebrae and intervertebral discs. The gradients visualization also indicates that the separability of vertebrae is one factor in the prediction. Different feature maps have activation at vertebral structures as well as more cohesive areas of the image. The activation maximization inputs show various patterns in several orientations suggesting a diverse set of learned feature extractors.

The learning curves (Fig. 7(b)) show good convergence with both training and validation error decreasing at the same rate for the first 20 epochs. The training curve continues to drop but the generalization ability of the model does not further improve. The model starts to overfit after 50 epochs of training.

When tested with the external KUH dataset, the models were able to detect unreliability with an average AUC of 0.94 (range 0.94–0.95) (Table 2). Test accuracy was 82.8%, sensitivity 92.7%, and specificity 72.9%.

### 3.3 Fracture prediction

**Fig. 5:**
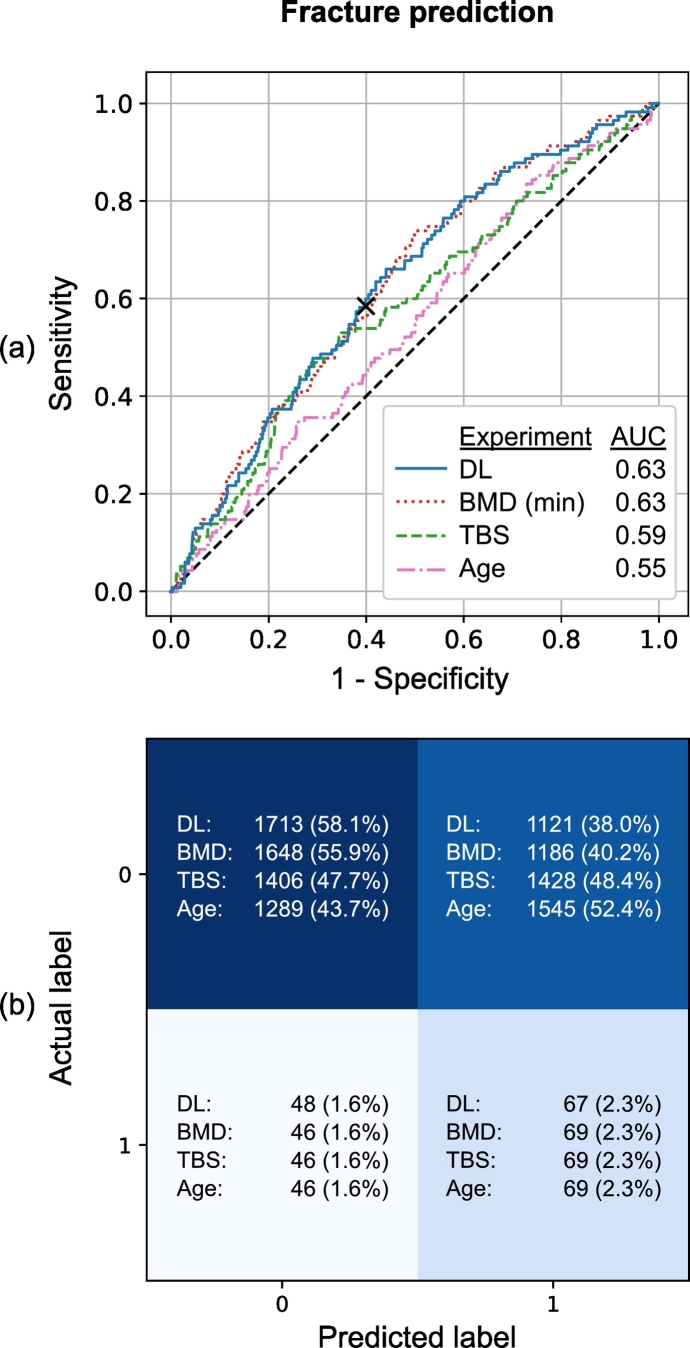
(a) Receiver operating characteristic (ROC) curves with the area under the ROC curve (AUC) values and (b) confusion matrices for fracture prediction taken from the OSTPRE dataset 10-fold cross-validation with the median AUC performance. The classification boundary of the deep learning (DL) model is marked in the ROC curve with an x-symbol. The figures also show the benchmark predictors of minimum BMD T-score, TBS, and age.

The fracture prediction produced an average AUC of 0.63. With the classification threshold adjusted to match the sensitivity of the minimum BMD T-score fracture predictor, the average accuracy was 52.0%, sensitivity 67.8%, and specificity 51.4% (Table 1 and Fig. 5). The benchmark predictors of the spine and hip T-score both produced average AUC values of 0.62. By using the minimum of these T-scores, the predictivity improved slightly (AUC 0.63). TBS performed with an AUC of 0.59 and age with an AUC of 0.55.

The logistic regression model with the minimum BMD T-score together with TBS and age did not improve the performance (AUC 0.63) compared to the minimum T-score alone. The minimum T-score combined with the prediction probabilities from the deep learning model improved the AUC to 0.64. The coefficient analysis showed that in this model the minimum T-score (*p* = 0.006) was statistically significant (*p* < 0.05) whereas the deep learning output (*p* = 0.158) was not. However, when the deep learning output (*p* = 0.041) was combined with the spine T-score (*p* = 0.028), they both remained significant. TBS did not improve the prediction and was not statistically significant when combined with any of the BMD T-score predictors or deep learning output.

**Fig. 6:**
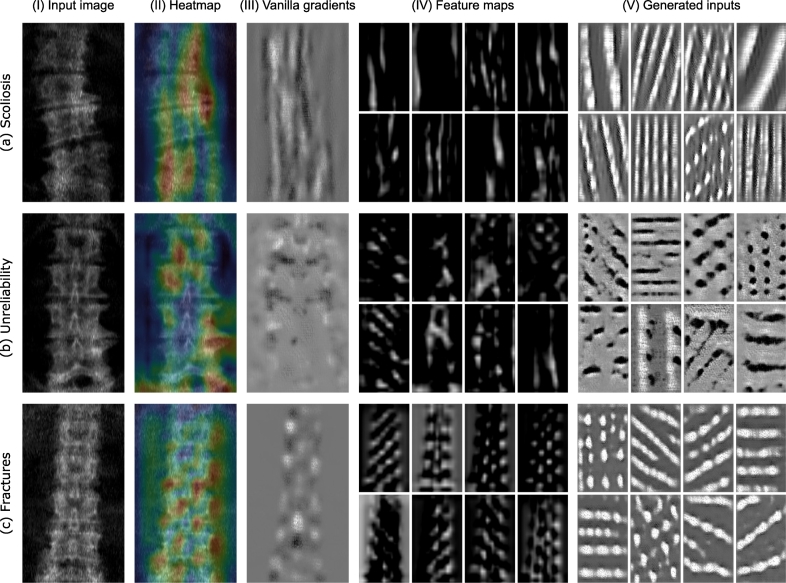
Visualizations of a correctly classified positive example case in (a) scoliosis detection, (b) unreliability detection, and (c) fracture prediction. (I) Preprocessed and cropped input image, (II) heatmap produced by gradient-weighted class activation after the last convolutional layer (red indicates the highest activation), (III) pixel-wise Vanilla gradients map (white indicates positive and black negative gradients), (IV) feature maps from randomly selected kernels (8 out of 64) of the last convolutional layer, and (V) generated inputs to maximize activation in randomly selected kernels of the last convolutional layer. Since the pixels that the DXA scanner produces are not square (0.6 mm of width and 1.05 mm of height), all the illustrations have been rescaled to appear anatomically correct.

The heatmap and gradient visualizations seem to focus on the vertebrae throughout the image area (Fig. 6(c) and supplementary material Fig. 10). The feature maps also show consistent patterns resembling vertebral structure. The activation maximization inputs appear somewhat blurry showing patterns in roughly horizontal orientations. This suggests that instead of extracting detailed features, the model is assessing the overall consistency of the spine structure.

**Fig. 7:**
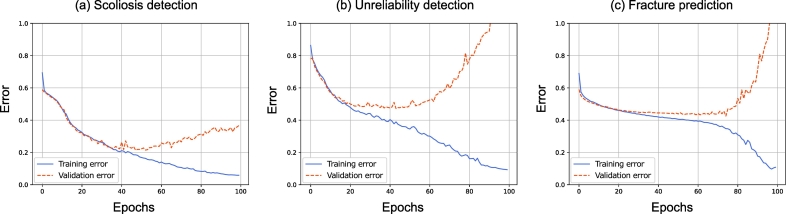
Learning curves of (a) scoliosis detection, (b) unreliability detection, and (c) fracture prediction taken from the OSTPRE dataset cross-validation subset with median AUC performance. The two curves for each case represent the average error of the training (blue) and validation (red) datasets over the course of training. The training was performed for 100 epochs, each epoch representing one pass through the entire training dataset. The model state in the lowest point of the validation error was restored at the end of the training.

The learning curves (Fig. 7(c)) show that the model slowly learns the optimal weights before heavy overfitting starts after 60 epochs. Further training fits the training data but is not able to generalize well on unseen samples. The overall decrease in the validation error is more restrained compared to the other classification tasks.

## 4 Discussion

In this paper, a deep learning approach to analyze spine DXA images was investigated. We conducted a series of experiments to find out if a convolutional neural network can be used to detect scoliosis and degenerative changes, and to predict fragility fractures. We also presented visual explanations of the predictions produced by the model.

With the OSTPRE dataset, the classification accuracy in scoliosis was 94.1% which exceeded the inter-rater agreement in radiologist labelling (92.5%). The AUC of 0.96 was higher than what was reported in a study detecting scoliosis in children from full-body DXA measurements (AUC 0.80) (Jamaludin et al., 2020). Interestingly, a study detecting scoliosis from back photographs (Yang et al., 2019) reported an AUC (0.95) close to our results. Scoliosis has an identifiable appearance and the visualizations suggested the network had learned a consistent and sensible recognition model. It appeared to be looking for bends from the profile of the spine. The failed predictions were largely borderline cases that pose difficulties also for human expert readers. Testing with the external KUH dataset led to a slight decrease in performance (AUC 0.93). This might be connected to the lower inter-rater agreement in the labelling compared to the OSTPRE dataset. However, the performance drop was small, which indicates that the model generalizes well to different data sources.

The performance in detecting unreliability was slightly lower (AUC 0.91), but the accuracy (82.4%) still surpassed the inter-rater agreement of the radiologists (78.8%). The closest reference found, a study detecting existing vertebral fractures from lateral DXA images of the spine (Derkatch et al., 2019), reported an AUC of 0.94. Although both studies were identifying structural anomalies, the formulation of the output label was different. Existing fractures have a more distinct appearance whereas our classification task included different types of degeneration and other abnormalities affecting image reliability. This difficulty was also observed in the visualizations (supplementary material Fig. 9). The model had often recognized areas of degeneration but could not always identify three or more sufficiently affected vertebrae. However, it was able to detect different kinds of abnormality, suggesting that the network had learned requisite feature extraction capabilities. The model generalized well to the external KUH dataset (AUC 0.94), even exceeding the validation results. The performance improvement was unexpected but could be explained by the differences in test population and labelling panel. The hospital data may have more bias compared to the population-based OSTPRE dataset. Also, the labelling of unreliability was a complex task and the quality of the labels could have improved as three experts were used instead of two. This seemed to produce more consistent results compared to the validation sets. Importantly, this result further indicates that the model generalizes well to different data sources and classification tasks.

The fracture prediction was the most challenging classification task. The deep learning model's AUC (0.63) slightly exceeded the benchmark predictivity of both lumbar spine BMD T-score (AUC 0.62) and hip BMD T-score (AUC 0.62) but was similar to the minimum BMD T-score taken from spine or hip (AUC 0.63). They all fall within BMD's typical fracture prediction AUC range of 0.60–0.75 reported in the literature (Leslie and Lix, 2014). The combined model of deep learning output and minimum BMD T-score produced the best AUC (0.64). This suggests that, in addition to bone density information, the deep learning model extracts other predictive information from the spine image. In our OSTPRE dataset, when predicting osteoporotic fractures, the neural network approach had a higher predictive ability than TBS (AUC 0.58). Interestingly, the deep learning model remained as an independent predictor of fractures when combined with BMD T-score from the lumbar spine, whereas TBS was not an independent predictor when combined with BMD T-score either from the lumbar spine or the hip, or with deep learning. Based on the visualizations, the deep learning model did not find very detailed features but looked for wider patterns that correlate with a good spinal condition, and apparently overall resistance to fractures.

The moderate level of the fracture prediction results was somewhat expected. Previously proposed models for fracture risk assessment have reported results at a similar level with little improvement to BMD (Ferizi et al., 2019; Goldshtein et al., 2018). The risk of fractures is complex and includes factors like the tendency to fall, which cannot be directly inferred from DXA images. This makes it difficult to estimate the highest attainable predictability. There is also some uncertainty involved in the fracture labels. Apart from the hip fractures, the information had been gathered by questionnaires. All the reported fractures were confirmed from medical records, but some occurred fractures may have left unreported (Honkanen et al., 1999). Furthermore, some minor vertebral fractures may not cause symptoms and therefore can go unnoticed.

The dataset used in this study was relatively small, so it represents only a subset of the real-world distribution. The limited size of the training set makes the model prone to overfitting as observed in the learning curves. This also limits the network architecture because deeper models generally require more training data. Data augmentation and regularization techniques can help to some extent, but they do not remove the need for comprehensive real-world samples, especially in the fracture prediction. It should be noted that only 92 patients (3.9%) had suffered fractures during the 5-year follow-up making the label heavily imbalanced. A longer follow-up period and an extended age range of participants could increase the proportion of positive examples. Another limitation is that the labelling for scoliosis and unreliability was based on only two experts in the OSTPRE dataset and three in the KUH dataset. As there is always some discrepancy in labelling between experts and even between labelling rounds, establishing the ground truth is difficult. For a more accurate assessment, a larger group of radiologists and other clinical experts should be incorporated.

The visualization results differed in all the classification tasks enabling us to reason about the feature extraction mechanisms. The revealed features seemed simple, which can be explained by the somewhat shallow architecture of the network. However, the visualizations should be interpreted with caution because the methods have their limitations (Rudin, 2019). For example, the heatmap methods have been shown to struggle when there are multiple contributing features in an image. Furthermore, in a deep learning model, the representation is distributed, and activations may need to be combined to form a meaning. No technique yet exists to explain a deep neural network completely.

## 5 Conclusion

In this study, we have found that deep learning can identify pathological features from DXA images at a level comparable to human experts. Incidental findings in bone mineral density measurements can indicate image unreliability and add information about the patient. We have also found that deep learning can extract features that predict fractures as accurately as bone density T-scores. Further research with larger datasets is needed to confirm the approach's generalizability and to reveal its full potential. However, we have shown that DXA images can be analyzed by using a robust convolutional neural network that works for different classification tasks. Deep learning could complement the current imaging standard for osteoporosis by gathering additional information from the image, and improve its predictive ability in the future.

## Declaration of competing interest

The authors declare that they have no known competing financial interests or personal relationships that could have appeared to influence the work reported in this paper.

## Appendix A Supplementary data

Supplementary material Image 1
