# Supplementary material for: Detecting pathological features and predicting fracture risk from dual-energy X-ray absorptiometry images using deep learning
Source: Bone Rep. 2021 Apr 24;14:101070. doi: 10.1016/j.bonr.2021.101070 (PMC8102403; doi:10.1016/j.bonr.2021.101070)
Supplement: Supplementary file 1 — Supplementary material [file mmc1.pdf]

Supplementary Material

Detecting pathological features and predicting fracture risk from dual-energy X-ray absorptiometry images using deep learning

Tomi Nissinen, Sanna Suoranta, Taavi Saavalainen, Reijo Sund, Ossi Hurskainen, Toni Rikkonen, Heikki Kröger, Timo Lähivaara, and Sami P Väänänen

1 Data preprosessing and augmentation

The dual-energy X-ray absorptiometry (DXA) images used in this study were produced with a Lunar Prodigy DXA scanner. The image data were exported from the DXA device in MATLAB binary format. The pixel intensities originally represented as integers were normalized across the whole dataset to zero-centred floating point values ranging from -1.0 to 1.0. Undefined pixels resulted from prematurely terminated scanning rows were converted to the minimum value of all scanned pixels.

The width of a DXA-image is standard 300 pixels, but the height varies depending on the length of the scanned region. In the original dataset, the total height range was 134 to 186 pixels. However, very few images had a height below 150 pixels (relating to an image size of 157,5mm), so that was decided as the minimum height for inclusion. Smaller images were dropped as anomalies and the rest were cropped to 150-pixel height from the top of the image. The DXA scan is usually extended well above the L1 joint, so this cropping did not significantly affect the region of interest.

The spine usually covers less than half the horizontal space in the image. Therefore, 150 pixels was also chosen as the target width. In some of the images the spine was not horizontally centred, so a simple centring algorithm was required to avoid cropping regions of interest. The centre of a spine was calculated by first summing the intensities column-wise, resulting to a row vector. Then the cumulative sum of the intensities in that row of pixels was calculated, and the half-point was determined as the centre line of the spine. The outcome of the algorithm was visually verified as sufficient for approximate centring.

Before training the neural network models, the used training sets were augmented by flipping. The corresponding pixel intensities from the left and right of the image were switched to create similar but mirrored additional training samples.

The pixels that DXA-scanner produces are not square but rectangular, 0.6 mm of width and 1.05 mm of height. The selected image size of 150 x 150 pixels corresponds to a real area of 90 mm x 157,5 mm. Therefore, the illustrations in the manuscript have been rescaled to correspond to the original pixel shape allowing the anatomies to appear correctly.

2 Model selection

The architecture for the manuscript was chosen after a careful experimentation process. We created several different models from scratch and searched for the best working parameters. Different combinations of layers, kernel settings, optimizers, and adjustable parameters were tested (Table 2). This process involved using Keras-tuner with Random search and Hyperband as well as further manual experimentation.

In addition to our custom models, we tested some widely used architectures, such as VGG, Inception, and DenseNet. Many state-of-the-art models proved too difficult to train for our input data and classification tasks. This is possibly due to their deep and complex architecture relative to our limited amount of data and small image size. The best performing ready-made models produced roughly similar results compared to our custom models (Table 3). By experimenting with the custom models, we found that adding convolutional layers beyond four did not significantly improve the results. Also, the adjustments in other hyperparameters had more effect on the stability and speed of the training process rather than the results. Many different custom model variants converged to similar results. The architecture containing four convolutional layers was selected for the manuscript due to its simplicity, reliable convergence, good training speed, and sufficient performance in all three classification tasks.

Table 2: Hyperparameter search values

Parameter	Value range / options
Kernel size	3x3, 5x5
Convolutional layers	2 - 10
Fully connected layers	1 - 3
Learning rate	1e-7 - 1e-2
Conv layer kernel count (per layer)	4, 8, 16, 32, 64, 128, 256
Dense layer node count (per layer)	16, 64, 128, 256, 512, 1024

Table 3: Model performance results

Model	Training speed (s/epoch)	Learning rate	Scoliosis AUC	Unreliability AUC	Fractures AUC
Custom model - 2 conv layers	1.56	3e-4	0.95 (0.95, 0.96)	0.86 (0.85, 0.86)	0.61 (0.59, 0.63)
Custom model - 4 conv layers	1.84	3e-4	0.97 (0.97, 0.97)	0.91 (0.90, 0.91)	0.63 (0.61, 0.65)
Custom model - 6 conv layers	2.26	3e-4	0.98 (0.97, 0.98)	0.91 (0.90, 0.91)	0.63 (0.62, 0.65)
Custom model - 8 conv layers	3.57	3e-4	0.96 (0.94, 0.98)	0.90 (0.90, 0.91)	0.60 (0.58, 0.61)
VGG16 (2015)	18.95	3e-5	0.97 (0.96, 0.97)	0.92 (0.91, 0.92)	0.64 (0.62, 0.65)
VGG19 (2015)	22.47	3e-5	0.97 (0.97, 0.97)	0.92 (0.91, 0.92)	0.63 (0.62, 0.65)
InceptionV3 (2016)	8.93	3e-6	0.80 (0.79, 0.82)	0.72 (0.69, 0.75)	0.61 (0.59, 0.62)
DenseNet121 (2017)	15.55	1e-6	0.87 (0.86, 0.88)	0.84 (0.83, 0.84)	0.59 (0.57, 0.61)

The Model performance results in scoliosis detection, BMD measurement unreliability detection, and fracture prediction using the OSTPRE dataset reported as the mean area under the ROC curve (AUC). Values shown are averages over 100 subsets (10 x 10-fold cross-validation) with the 95% confidence intervals in parenthesis. All the reported custom models used a kernel size of 3x3 and had a single fully connected layer with 256 nodes. The kernel counts started from 8 in the first convolutional layer and gradually increased to 64 towards the last layer. Dropout (rate 0.5) and L2 regularization (rate 0.003) were used in the custom models. The models were trained with binary cross-entropy loss, Adam optimizer, and batch size of 256 (except DenseNet with 64). The learning rates and training speeds are reported in the table.

3 Additional visualization results

3.1 Scoliosis detection

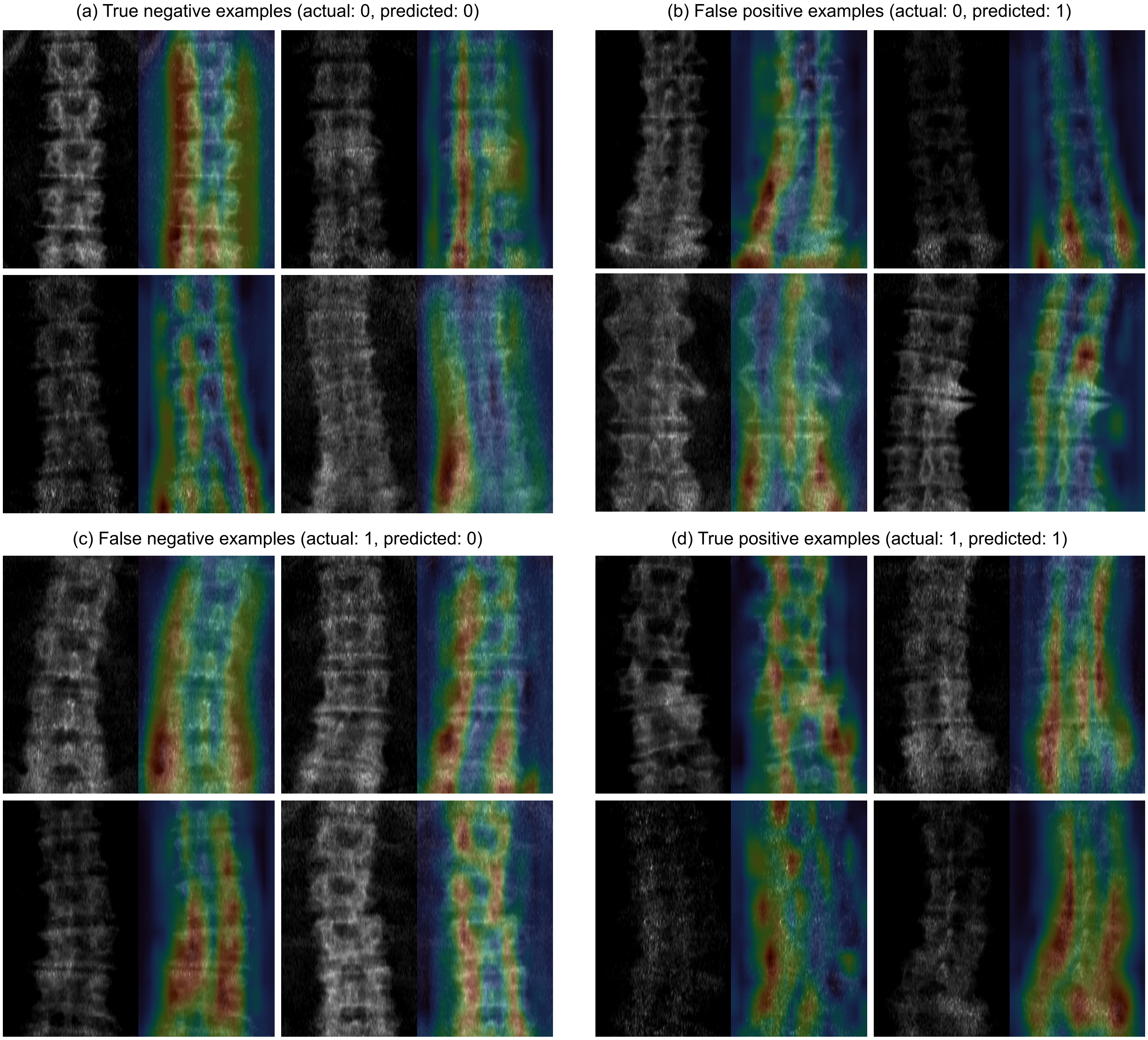

Figure 8: Examples of input images and prediction heatmaps in scoliosis classification. (a) Four cases where the model correctly predicted the negative (no scoliosis) class. (b) Four cases where the model incorrectly predicted positive when the actual class was negative. (c) Four cases where the model incorrectly predicted negative when the actual class was positive. (d) Four cases where the model correctly predicted the positive class. The heatmaps suggested that a uniform curve or line of high intensities was associated with scoliosis or no scoliosis.

3.2 Unreliability detection

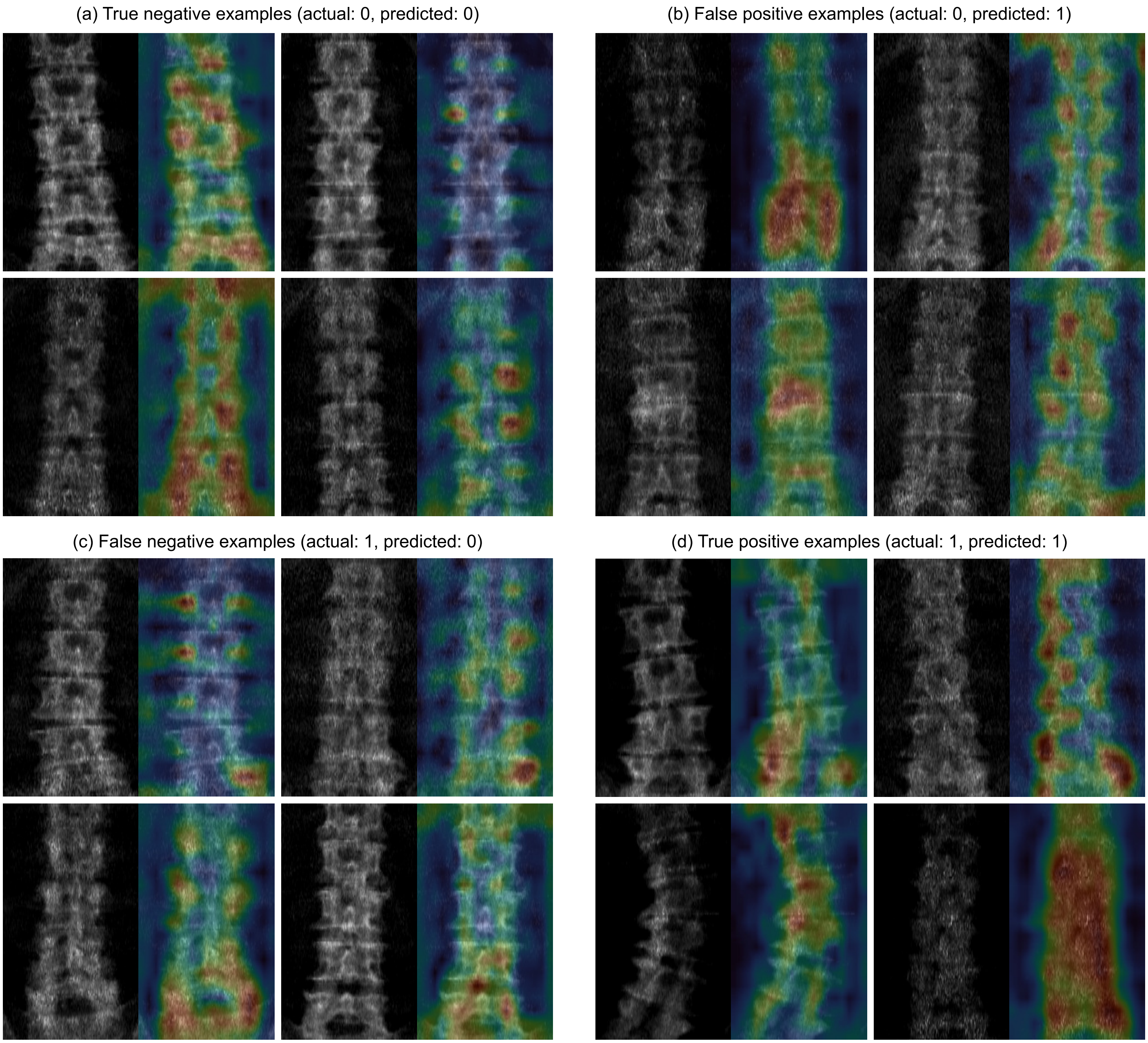

Figure 9: Examples of input images and prediction heatmaps in unreliability classification. (a) Four cases where the model correctly predicted the negative class (image is reliable). (b) Four cases where the model incorrectly predicted positive (is unreliable) when the actual class was negative (is reliable). (c) Four cases where the model incorrectly predicted negative when the actual class was positive. (d) Four cases where the model correctly predicted the positive class. The heatmaps are more heterogeneous in unreliability classification than in scoliosis. In false positive and false negative cases maximum intensities of the heatmaps are more often scattered asymmetrically in the pedicles.

3.3 Fracture prediction

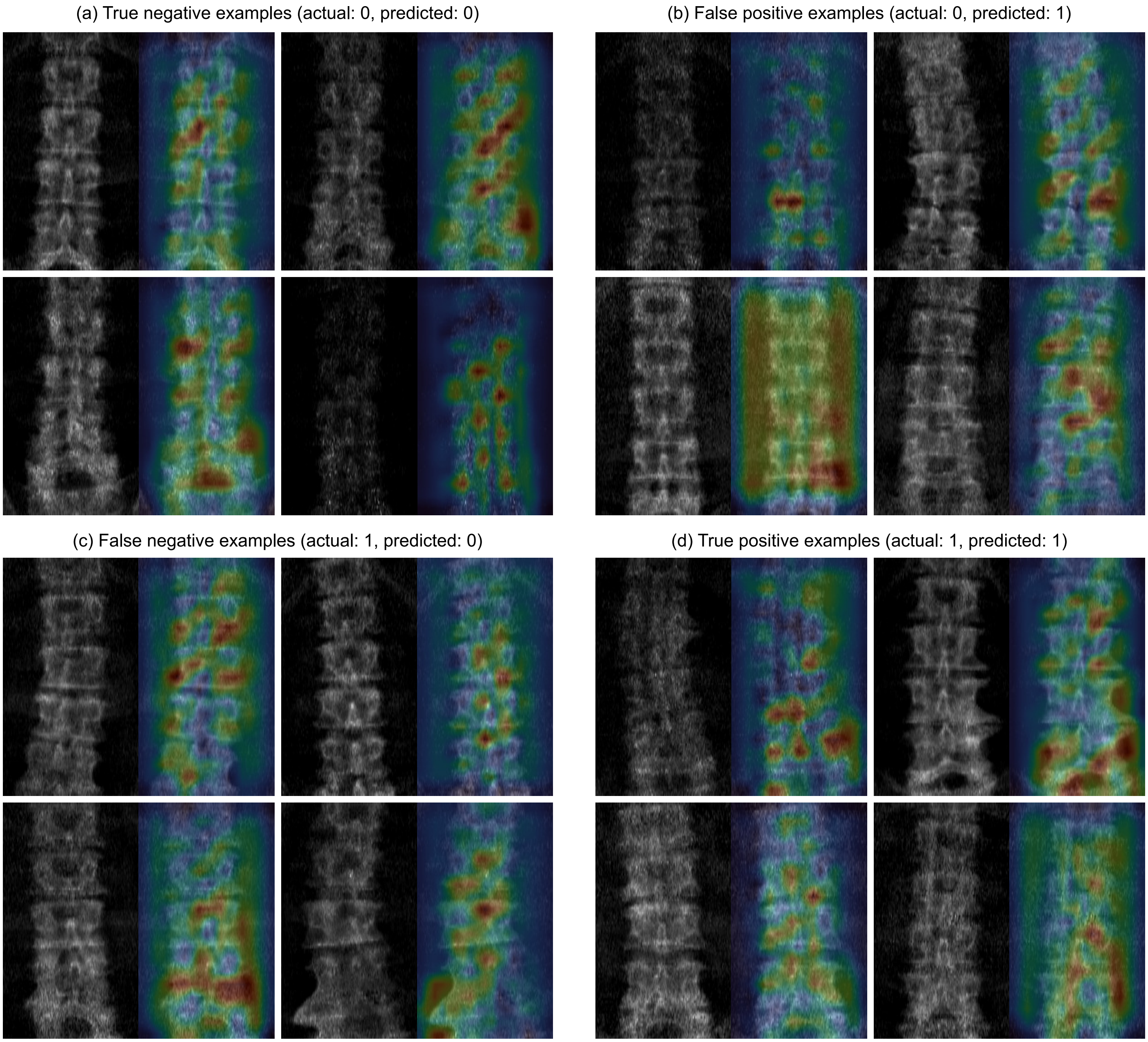

Figure 10: Examples of input images and prediction heatmaps in fracture prediction. (a) Four cases where the model correctly predicted the negative class. (b) Four cases where the model incorrectly predicted positive when the actual class was negative. (c) Four cases where the model incorrectly predicted negative when the actual class was positive. (d) Four cases where the model correctly predicted the positive class. The heatmaps suggest that the model is evaluating the consistency of the vertebrae throughout the image area.
